# Software architecture for pervasive critical health monitoring system using fog computing

**DOI:** 10.1186/s13677-022-00371-w

**Published:** 2022-11-30

**Authors:** Abeera Ilyas, Mohammed Naif Alatawi, Yasir Hamid, Saeed Mahfooz, Islam Zada, Neelam Gohar, Mohd Asif Shah

**Affiliations:** 1 Department of Computer Science, University of Peshawar, Peshawar, Pakistan; 2 Information Technology Department, Faculty of Computers and Information Technology University of Tabuk, Tabuk, Saudi Arabia; 3 Abu Dhabi Polytechnic, Abu Dhabi, United Arab Emirates; 4 Faculty of computing, International Islamic University Islamabad, Islamabad, Pakistan; 5 Shaheed Benazir Bhutto Women University, Peshawar, Pakistan; 6 Kebri Dehar University, Kebri Dehar, Ethiopia

## Abstract

Because of the existence of Covid-19 and its variants, health monitoring systems have become mandatory, particularly for critical patients such as neonates. However, the massive volume of real-time data generated by monitoring devices necessitates the use of efficient methods and approaches to respond promptly. A fog-based architecture for IoT healthcare systems tends to provide better services, but it also produces some issues that must be addressed. We present a bidirectional approach to improving real-time data transmission for health monitors by minimizing network latency and usage in this paper. To that end, a simplified approach for large-scale IoT health monitoring systems is devised, which provides a solution for IoT device selection of optimal fog nodes to reduce both communication and processing delays. Additionally, an improved dynamic approach for load balancing and task assignment is also suggested. Embedding the best practices from the IoT, Fog, and Cloud planes, our aim in this work is to offer software architecture for IoT-based healthcare systems to fulfill non-functional needs. 4 + 1 views are used to illustrate the proposed architecture.

## Introduction

Medical informatics, healthcare, and Internet health technologies are all combined in the cutting-edge system known as e-health. This combination encourages technological advancements to address persistent issues like lower costs and higher-quality healthcare. Likewise, the Internet of Things (IoT) enables a wide range of innovative initiatives to be implemented with shared and programmable technologies such as the Internet, cloud computing, storage, network-connected gear, software, and databases. When combined with adaptable, easily accessible, and enhanced patient health services, IoT and cloud computing has developed into revolutionary inventions that strengthen one another’s capabilities. This connection is easier to adopt in comparison to traditional networks, makes information more secure during engagements, increases data access, and increases productivity. Healthcare services can be considerably improved by IoT and cloud-based e-Health systems, which also encourage continuous, methodical development. IoT and cloud-based e-Health platforms allow users, providers, and servers to interact with each other while maintaining medical data in the cloud [1].

On the other side, there are numerous important problems with cloud computing, including traffic overload, huge data processing volumes, and data transmission delays. The main cause of these difficulties is that cloud servers are physically far away from IoT devices [2]. Healthcare apps cannot tolerate delays due to the critical nature of the industry. Employing specific cloud computing services to collect and analyze medical data from patients dispersed across a large geographic area is not practicable due to the significant transmission latency and high network utilization [3]. Fog computing has become a paradigm change to address the fundamental issues with conventional cloud computing as mentioned above [4]. As a component of the fog computing architecture, a wide variety of devices are linked to the network to supply computational and storage resources. Fog computing provides an infrastructure that is more flexible, and secure, and requires lesser bandwidth. The idea of healthcare systems reinforces that, in most countries, healthcare has challenges that only worsen with the aging population [5].

Health monitoring systems and Internet of Things advancements in wireless and cellular networks greatly enhance performance and save medical costs. Providing inexpensive home monitoring equipment which can spot early signs of deterioration, prompt service and treatment can be provided. IoT encourages observation of critically ill patients. Moreover, in developing countries, the population is mostly residing in rural areas where medical assistance is not easily available. Also in disaster-stricken areas, a framework is needed which can assist critical patients remotely using existing infrastructure. As a result, a framework for IoT-assisted health monitoring based on fog computing is suggested. The design of the suggested health monitoring system provides patients with ongoing real-time medical response, and fog computing has demonstrated its effectiveness in time-sensitive applications. Fog computing provides a way to handle the massive amounts of data created by end-user devices by deploying resources close to ending users. Due to the requirement for quick and effective data processing, fog computing should thus be employed in the scenario that has been presented.

The presented research is a bidirectional approach to improve real-time data transmission for health monitors by reducing network latency and usage. To that end, a simplified approach for large-scale IoT health monitoring systems is devised, which provides a solution for IoT device selection of optimal fog nodes to reduce both communication and processing delays. Additionally, an improved dynamic approach for load balancing and task scheduling is also suggested. Embedding the best practices from the IoT, Fog, and Cloud planes, our aim in this work is to offer software architecture for IoT-based healthcare systems to fulfill the aforementioned non-functional needs.

However, the computing and storage capacities of fog servers are constrained. The load on the fog server increases as the number of available queries rises in a vast system [6]. Patient requirements to install the proposed fog-based health monitoring system globally will put pressure on a single fog node. The fog node in this case gets overloaded while the other fog nodes are most likely dormant, increasing response time and causing the delay. Moreover, the selection of an inappropriate fog node for the IoT device gateway to transmit data increases network latency.

The three architectural layers that are suggested include sensors that are attached to the patients and can detect and transmit vital signs like body temperature, heart rate, and pulse rate to the IoT device gateways in the first tier. The fog servers, which are located within the same area as the base stations, make up the intermediate fog layer (BS). The fog nodes receive the real-time monitoring data from the first tier’s IoT devices, process it to determine whether the patient is in a critical condition, and then transmit their findings to the cloud server through a proxy server set up in the third layer. The fog nodes also notify the patient’s PDA devices of the results of their health status monitoring. Fog computing provides a way to handle a sizable volume of data produced by end-user devices by bringing resources close to end-users. Fog computing is therefore appropriate for the recommended technique because real-time efficient data processing is required. However, due to the pressure placed on fog nodes and the scanning of the IoT gateway for a suitable fog node to relay monitoring data, transmission is prone to delays. The IoT sensor gateway experiences a communication delay (D_c_) as a result of the time needed to connect with an appropriate fog node, which renders it inappropriate for real-time monitoring. Additionally, the burden on the fog nodes increases the processing time (D_p_), slowing transmission. To increase the quality of service (QoS), both network delays should be addressed. The overall delay can be written as,

$$\mathrm{Total}\;\mathrm{delay},\;{\mathrm D}_{\mathrm t}={\mathrm D}_{\mathrm c}+{\mathrm D}_{\mathrm p}$$

To manage the total network delay, this research suggests an innovative two-way method in the suggested architecture, which manages the incoming monitoring traffic at fog nodes. Firstly, by connecting to the optimum fog node, an efficient scanning mechanism (ESM) for the IoT gateway suggests a method to minimize communication lag. Secondly, we present a network-assisted Load Balancing scheme for real-time monitoring data (LBRT). The strategy effectively distributes the load to nearby fog nodes to reduce latency and network utilization because the health monitoring system’s time-sensitive nature makes it necessary. As we know, IoT data flows experience traffic and processing latency, as described in [7]. The frequent data transmission to the cloud server is reduced by using fog-based computing in the system design, thus lowering the system’s latency. The following are the contributions of this paper:

1. A three-tier architecture is proposed for the fog-IoT health monitoring system, with fog nodes located in the middle layer. Smartphones connected to body sensors relay the patient’s physiological data streams to the fog node. To determine whether a patient’s health status is critical or not, fog nodes process the incoming data streams. The patient’s health information is thereafter sent to their smartphone and sent to the cloud for storage.
2. In widely deployed health monitoring systems, the load-balancing of the real-time data scheme (LBRT) balances the load among fog nodes.
3. The IoT gateway is suggested to use the efficient scanning mechanism (ESM) to rapidly and effectively choose a suitable fog node for transmission.
4. Two criteria, namely latency and network utilization, are used to assess the performance of LBRT for health monitoring.
5. Using the iFogSim toolkit, extensive simulations are performed to compare the performance of the LBRT to the conventional fog node placement method (FNPA) [8], and the load balancing scheme (LBS) [9].

The following is how the paper is set up: The context and inspiration of this work are presented in Section II. The most recent research on load balancing in fog-based systems and the design of health monitoring systems is presented in Section III. The proposed architecture for health monitoring systems is detailed in Section IV, and the real-time (LBRT) load balancing algorithm is covered in Section V. The discussions in Section VI and Section VII provide the conclusion and suggestions for future development.

## Related work

There are various architectures presented by scholars depending on the scenario. Healthcare organizations using IoT have been encouraged to mobilize using a sustained security plan (SSP), according to [5]. The suggested strategy calls for developing a reliable and effective architecture for DTLS certificate-based end-user license authentication and smart gateway-based mobility support. The study [10] presents a detailed architecture of the IoT-based healthcare ecosystem and examines the application of IoT in healthcare (CAHE). According to Article [11], medical devices are linked to an intelligent one-to-one oneM2M system. In their proposed system, the protocol between ISO/IEEE 11073 protocols and oneM2M protocol messages is transformed at the gateways between medical equipment and the Personal Healthcare Device (Ph.D.) management server. Resulting of the tests, it can be concluded that protocol conversion was successful. According to [12], a cloud-based healthcare diagnostic framework (CHDF) and an Internet of Things-based system can predict potential fatal illnesses based on diseases. Measurement, accuracy, sensitivity, and specificity were used to calculate the results. Developing an Intelligent Health Monitoring Architecture (IHMA) with this research was the goal of [13]. To demonstrate the use of massive data in healthcare, the authors examined, evaluated, analyzed, and tested several types of data.

Using drones in healthcare and connecting them to cloud-based infrastructure, remote servers, or body area networks (BANs) is part of medical IoT, according to [14]. For the collection, processing, and sharing of patient data in real-time, [15] proposes a Patient-based Architecture (PA). Information about patients can be transmitted using this architecture under time constraints. Two case studies were discussed to evaluate the performance of this architecture.

Doctors would be under additional pressure as the amount of data gathered through patient monitoring devices and the number of analytical choices made to acquire various data kinds for each patient increased. In [16], a suggested WBAN-based IoT healthcare system is examined together with IoT-based healthcare applications. It also does a real-time analysis of authentication, energy, power, resource management, quality of service, and wireless health monitoring—features that are extremely troublesome in terms of security and privacy. The authors of [17] presented an architectural design for all-encompassing healthcare systems. They formalized their modeling process and used model-checking tools to validate attributes like dependability and availability. As part of their review of the health monitoring system in a smart environment from the viewpoint of the public, the authors of [18] described the current state of intelligent health monitoring systems. Based on the authors’ analysis of technology development, requirements, design, and modeling, these systems face challenges. A 6G-aware fog federation architecture is developed in [19] to utilize fog resources, meet the need for specialized services, and ensure the shortest possible service delay and cost. A hierarchical fog network is used in [20] to manage the energy performance tradeoff while scheduling and offloading real-time IoT applications using the Energy-Efficient Task Offloading (EETO) policy. Additionally, we developed a stochastic conscious data offloading problem with a successful virtual queue stability strategy based on a heuristic method for allocating priority to each incoming job. Using current status data, the proposed method reduces queue waiting times and energy consumption. Based on simulations with various QoS parameters, it appears that the proposed EETO mechanism is more energy-efficient and works better. Based on the gateway’s advantageous position at the edge of the network, a variety of services were delivered, including local storage, local data processing, and embedded data extraction. An intelligent smart gateway prototype, UT GATE, was presented by the authors in [21]. IoT-based health monitoring systems are expected to increase energy economy, mobility, performance, interoperability, security, and dependability, based on proof-of-concept designs. As described in the publication [22], deep learning is integrated into Fog to be used as a useful technique for investigating heart disease. The framework is called Health Fog. This framework provides health services, such as Fog services, with an emphasis on heart-related illnesses. The effectiveness of this approach is assessed using attributes like power usage, network bandwidth, delay, and response time are used to gauge this framework’s performance. To facilitate the process of data conditioning, intelligent filtering, smart analytics, and selective transmission to the Cloud for long-term storage and temporal variability monitoring, a Smart Fog Gateway (SFG) model for end-to-end analytics in wearable IoT devices are provided in [23]. The execution time and energy consumption of this model are both optimized for performance. The work in [24] proposes an innovative Healthcare Monitoring Framework (HMF) based on a big data analytics engine and a cloud environment to accurately store and assess healthcare data and to increase categorization accuracy. The findings demonstrated that the suggested model accurately manages heterogeneous data, enhances the classification of health conditions, and improves drug side effect predictions. In [25], an evacuation system (CFaES) based on the Internet of Things and cloud-fog technology is suggested. The Fuzzy K-Nearest Neighbor (FK-NN) approach is used by the system to analyze the panic health state in real-time using the Fog computing paradigm. Experimentation demonstrates the suggested system’s effectiveness at various levels.

For healthcare services, a general Edge-of-Things Computation (EoTC) paradigm is put out [26] to reduce costs associated with resource supply and data processing. Performance evaluation using QoS parameters is missing from this framework [27]. presents an event-driven IoT architecture with context, event, and service layers for data analysis of dependable healthcare applications. Several layers take into consideration dependability factors, and the Complex Event Processing method incorporates automated intelligence and a revolutionary approach. As part of the Healthcare Big Data Analytic architecture (SLA-HBDA), Service Level Agreements (SLAs) are employed to rank patient data [28].

An efficient data distribution strategy is developed using the Spark platform to disseminate batch and streaming data to predict the patient’s health state. When compared to the Naive-Bayes (NB) algorithm, the SLA-HBDA architecture performs better in terms of accuracy, but it ignores latency and other crucial QoS factors [29]. proposes an IoT-based system for classifying streaming data according to the level of criticality. This framework computes the critical data in the fog to quickly and accurately detect anomalies. The model presented by [17] utilizes green energy and reduces latency and power consumption in secure IoT systems by using multisensory frameworks. To handle large numbers of requests and their quality and security limitations, a Genetic Algorithm is proposed. Compared to a baseline approach, the proposed strategy reduces edge device delay and power consumption simultaneously. According to the type of traffic, a suggested architecture in [30] will forward data to the cloud or fog layer. To effectively tackle this problem, load balancing is used to choose the best fog or cloud to convey the data from the device. The suggested effort focuses on decreasing packet and delay. A four-tier design was suggested in [31] to identify the specific decision-maker for job offloading. The authors abstracted this issue as a population game that Maynard replicator dynamics can resolve. An overview and recent developments of digital twins for healthcare 4.0 were described in [32]. Also suggested is a digital twin architecture for the healthcare industry. They also provided several digital twin use cases and open research problems with potential solutions [33]. focused on the state of the art, the enabling technologies for implementing the Metaverse for healthcare, the prospective applications, and the connected initiatives while providing a thorough assessment of the subject. As part of future research paths, the problems with the Metaverse’s adaption for healthcare applications are also emphasized, along with some likely fixes. Table 1 shows the literature review of the related work.

**Table 1:** A literature review of related work

S. No	Them of the paper	Observations
[5]	DTLS certificate-based end-user license authentication and smart gateway for mobility	The suggested strategy calls for developing a reliable and effective architecture for DTLS certificate-based end-user license authentication and smart gateway-based mobility support but has latency and power consumption problems.
[10]	IoT-based healthcare architecture (CAHE)	Having bandwidth problem.
[11]	message-based one-to-one intelligent M2M system for medical and PHD devices	Device-to-device messaging techniques have delay problems.
[12]	cloud-based diagnostic framework (CHDF) assisted by IoT	Provides limited resources to the end users.
[13]	an intelligent health monitoring architecture (IHMA	Delay and latency problems
[14]	using drones in cloud-based health infrastructure along with remote servers and BANs for medical IoT	Security and sensitivity issues.
[15]	Patient-based architecture for real-time medical data handling	Based on only two case studies.
[16]	WBAN-based IoT healthcare system architecture for real-time analysis in terms of authentication, energy, and QoS	Delay, latency, and power consumption problems
[17]	an all-encompassing architectural design for healthcare systems	Formalized their modeling process and used model-checking tools to validate attributes like dependability and availability
[18]	the current state of the intelligent healthcare system in a smart environment -a public view	Based on the authors’ analysis of technology development, requirements, design, and modeling, these systems face challenges
[19]	6G aware fog federation architecture measuring cost and delay	No security mechanism is included
[20]	Energy-efficient task offloading (EETO) based on hierarchical fog architecture to manage energy by a tradeoff	Resources allocation and time scheduling problems.
[21]	an intelligent smart gateway (UTGATE) for IoT-based health monitoring systems using proof of concept	Delay and latency problems
[22]	deep learning supported the analysis of heart disease using Health Fog	Energy consumption is ignored.
[23]	Smart Fog Gateway (SFG) model for end-to-end wearable IoT devices	Authentication problems may occur.
[24]	Healthcare monitoring framework (HMF) based on big data analytics in a cloud environment	Bandwidth and delay problems may occur.
[25]	an evacuation system (CFaES) based on IoT and cloud-fog using Fuzzy KNN	Cost and time delay problems may occur.
[26]	Edge of things computation (EoTC) model is used to minimize resource supply and data processing	Performance evaluation using QoS parameters is missing from this framework
[27]	event-driven IoT architecture for dependable healthcare applications	Problems may occur in the real-time data processing
[28]	SLA-HBDA model is developed to rank patient data for analysis	It is based on only one parameter and it ignores latency and other crucial QoS factors
[29]	The IoT-based system is used for classifying streaming depending on the criticality	Factors like Time delay and latency are ignored.
[17]	a generic algorithm-based energy-efficient model for a multisensory secure IoT system	A Delay problem may occur.
[30]	traffic type-based architecture for a decision on data handling by fog or cloud	Ignore crucial factors like security and bandwidth.
[31]	based on a 4-tier design to identify job offloading factors.	Security and delay problems may occur.

## Proposed architecture

This section illustrates the three-tier architecture of a fog-based health monitoring system, which is like the approach described in [9]. In the first tier of the suggested architecture, the critical patient wears sensors to monitor vital signs including body temperature, heart rate, pulse rate, and other vitals, which are then transmitted to the fog nodes via telephones. Fog nodes make up the second stratum of the design. The fog nodes, which are placed next to the BSs, analyze the data collected from IoT devices. The patient’s smartphone then receives the results of their health state. The fog nodes are positioned closer to the IoT devices at the network’s edge to guarantee a quick reaction in a real-time setting. Detailed layers are discussed below.

**Fig. 1:**
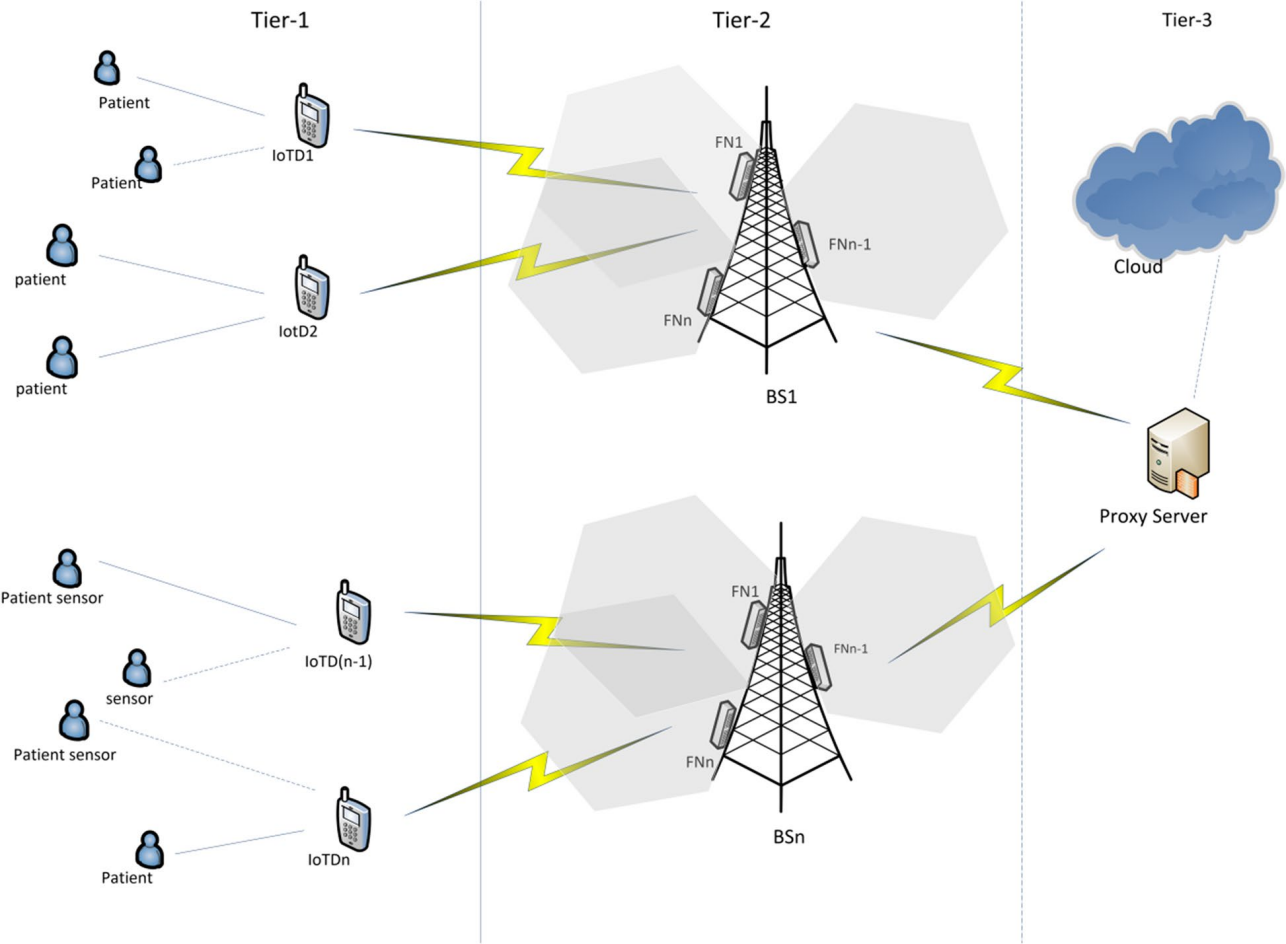
General schema of the proposed model

As seen in Fig. 1, the proposed schema comprises three tiers.

### Tier 1 (device layer)

Smart medical devices based on IoT are included in this layer, allowing users to check their health anywhere, anytime. Medical technologies such as blood pressure, ECG, and sleep monitoring can be used in this setting to monitor the health of elderly patients 24 hours a day. Another important module component in this layer is the IoT device gateway (IoTDW). It is a PDA device that connects the body sensors with the BS. The IoTDW receives the real-time monitoring data from the body sensors and interprets them accordingly to be delivered to the fog nodes that are co-located at the BS. As the data deals with real-time data with the same priority, an efficient scanning mechanism is deployed in the scanning module component of the IoTDW.

- *Data transfer (Connectivity):* The ability to periodically transmit medical data gathered from sensors to the lower layers while also receiving messages and data from the cloud and fog layer.
- *Device management:* The sensors must frequently send the monitoring data to the IoT device gateways for real-time processing.
- *Notification (Alert notification event):* The PDA is capable of receiving any notification from the fog nodes in case of emergency to alert about the emergency.
- *Connection Establishment:* The scanning module utilizes the BS_SL_ list generated by BS. It allows the IoTDW to take an informed connection establishment decision.

### Tier 2 (fog layer)

For extremely important instances, the Fog Layer of the proposed healthcare platform reviews time-sensitive data and takes a real-time decision. The fog layer lies comprises BS and the co-located fog nodes. As previously said, the purpose of the fog layer is to reduce the amount of time spent calculating, storing, and processing data while maximizing mobility and availability while achieving high reliability and efficiency. Nodes of this layer were regarded in all earlier studies as smart gates that carried out different functions. However, considering the importance of these systems, it may be difficult to employ mandatory and insufficient demands at this level due to costs. Some advantages of using the proposed framework of the device and fog layer include:

- BS involvement to reduce the burden on Fog nodes for task management
- Reducing network traffic
- Dynamic allocation of tasks to the fog nodes when the threshold reaches
- BS stat list (BS_SL_) makes the whole framework fault tolerant.

Below is a discussion of some fog layer characteristics

- *Local Storage:* The fog layer consists of multiple fog nodes attached to a BS. Each fog node holds its storage to maintain the input data temporarily.
- *Consistency:* The fog layer must always be adaptable and compatible, especially in the event of an emergency. Dynamic reconfiguration of developed machine learning algorithms can be employed for various services [10, 13]. Consistent data transfer is required in the fog layer. As part of this process, both sensors request data and the fog layer assesses the data received from the cloud. The system must have recognized an increase in priority level when the irregularity occurs in long-term cardiovascular patient observation. Data transport to the Cloud is also given priority for many characteristics and services. Emergency patients must have a higher priority for data transmission to the cloud than chronic patients with slower transfer rates [34].
- *Local processing:* Data analysis at the fog layer increases system sensitivity. By recognizing and anticipating emergencies, the system can respond more quickly. Rather than transmitting parameters to the cloud and waiting for a response, the fog layer can detect strokes locally if elders are at risk for serious conditions (such as strokes). Consequently, the system will be able to respond more accurately and handle problems faster. By leveraging data analysis at the fog layer, the system can also shorten processing times for critical metrics. Through the analysis of local data and feedback from local users, the system’s availability and reliability can be increased even without access to global data.
- *Database:* For later retrieval and processing, the gateways store incoming data in the local database of the Fog layer. The type and relevance of the data will determine whether it should be compressed or encoded. Data archiving at the fog layer boosts efficiency for ongoing data storage due to network constraints while transferring data to the Cloud and processing by fog nodes.
- *Scheduling:* It is crucial to respond to these requests and allocate resources as efficiently and quickly as possible. Several occurrences and requests are transferred to electronic healthcare systems at once. The task assignment module in fog nodes provides a mechanism to evenly distribute the incoming tasks among fog nodes. In this way, not only a specific fog node will not be overburdened but other fog node resources will also be utilized in a distributed manner. The suggested approach for this is dynamic in way that it assigns tasks considering the current load status of the fog node. In this way, by offering a top-notch workflow scheduler, numerous non-task requirements, such as availability, performance, and reliability, can be improved.

### The tier 3 (cloud layer)

**Fig. 2:**
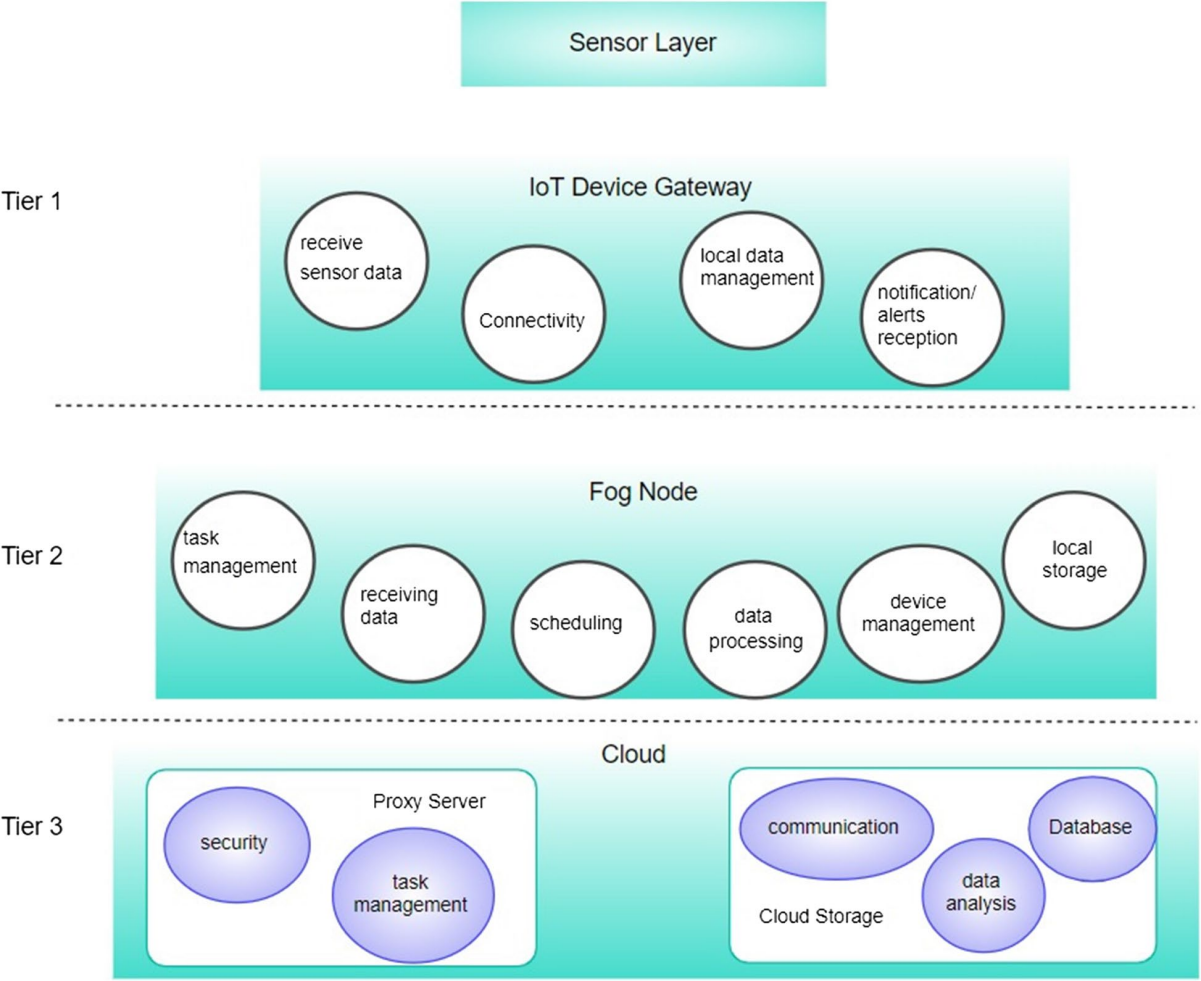
Layered Architecture of proposed framework

This layer exchanges information with the Fog layer and also receives processes and stores data. Because these systems (physicians, patients, etc.) seamlessly integrate with other resources such as electronic versions and web resources, users (physicians, patients, etc.) can access the information whenever and wherever they need it. The platform’s device and cloud layers are separated, which improves application flexibility and, generally, makes healthcare apps more effective. It is possible to classify patient-related health data as either public or private using the public and private Clouds in this layer [10]. Processing the patient’s medical data in the cloud layer, gives insights into the present health status, thus, providing support to medical practitioners in taking a decision. The cloud layer architecture is depicted in Fig. 2. Each cluster in this system has a proxy server, which interacts with a controller over the network. To keep the delivery of clustered jobs on schedule, the controller must create a component in this regard.

## Abstract models of the proposed architecture

Crouch developed the 4 + 1 model (Fig. 3) to explain the architecture of software systems.

**Fig. 3:**
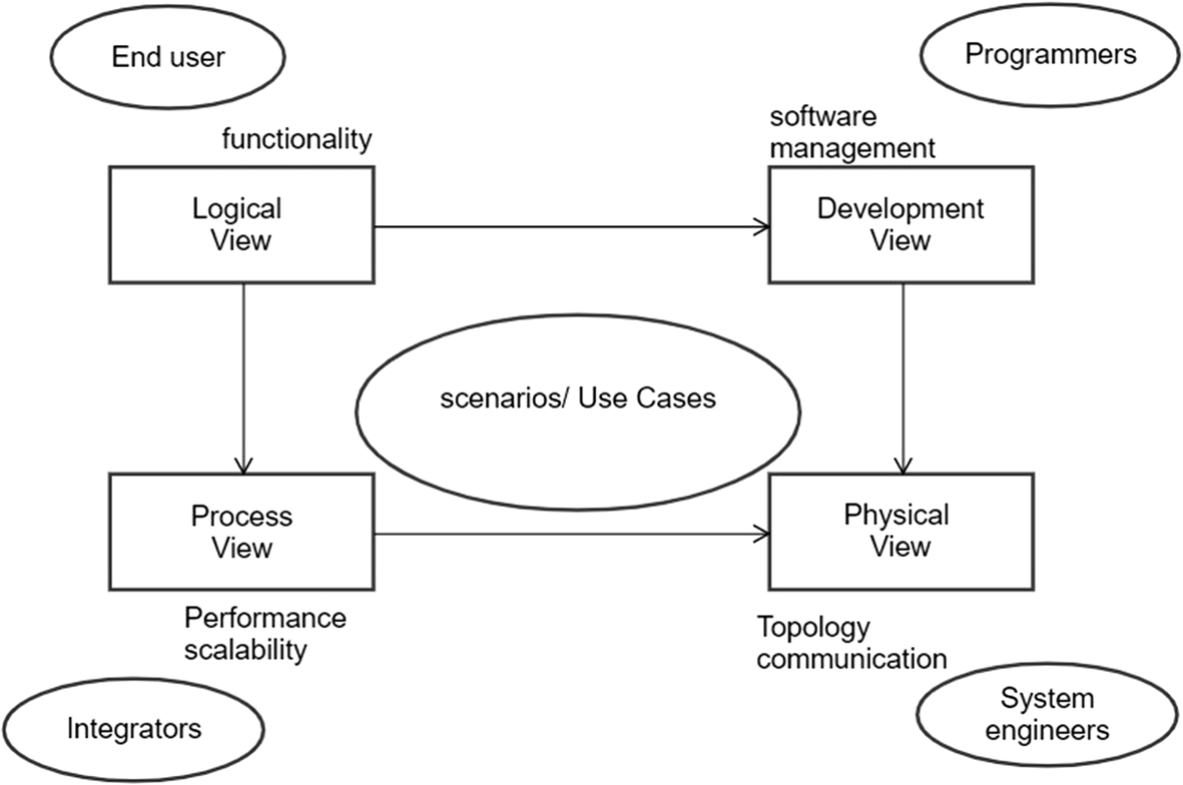
4 + 1 model for software systems

- *Logical View*: The system’s features are provided to users as a part of the logical perspective. Diagrams of classes and steps are used to illustrate the logical view. The main sequence diagram for our system, shown in Fig. 4, shows how the parts work together.

**Fig. 4:**
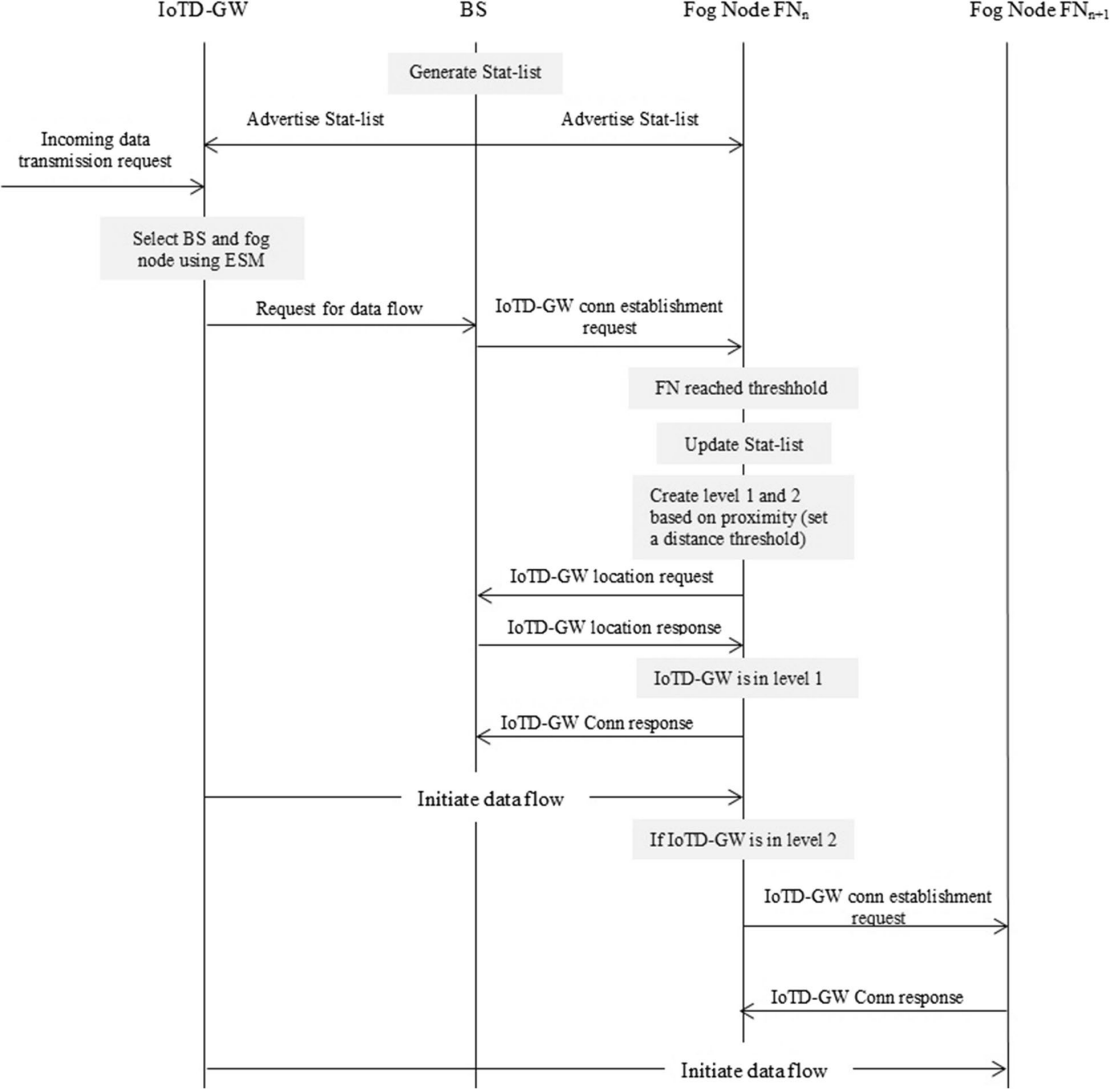
Main sequence diagram

- *Development View:* Components of the proposed model can be identified using UML. The proposed architecture’s component diagram is shown in Fig. 5:
- Component on the IoTDW:

i. Receiving monitoring data from sensors.
ii. Connection with Fog layer
iii. Receive alerts
iv. Manage local data
v. Interface

- Component of fog nodes:
  i. Receiving data from IoTDW.
  ii. Device manager
  iii. Scheduling
  iv. Task assignment
  v. Data processing
  vi. Local data storage and retrieval

- Component on the cloud:

i. Data storage
ii. Data analysis
iii. scheduling

**Fig. 5:**
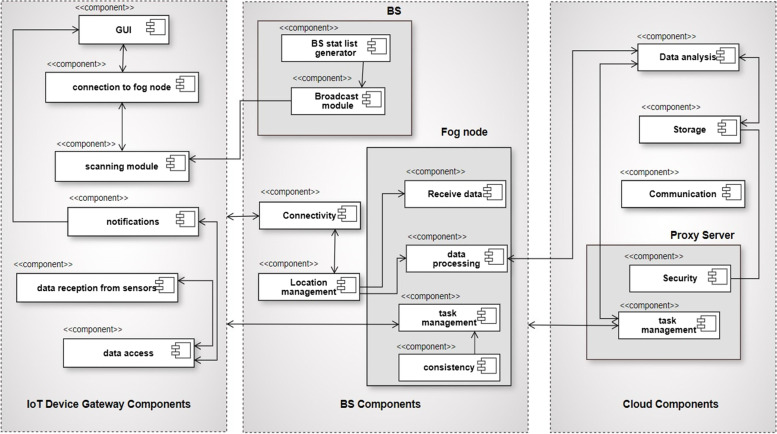
Component diagram

*Process View:* A process perspective talks about the system’s dynamic elements and explains its processes and how they interact. It also emphasizes how the system behaves while it is running. Concurrency, distribution, performance, and scalability are all covered by the process view. When the Fog node detects an emergency and contacts the closest medical facility via the gateway, the data will be saved. If necessary, the patient’s doctor or family will also be contacted. Figure 6 depicts this scenario.

**Fig. 6:**
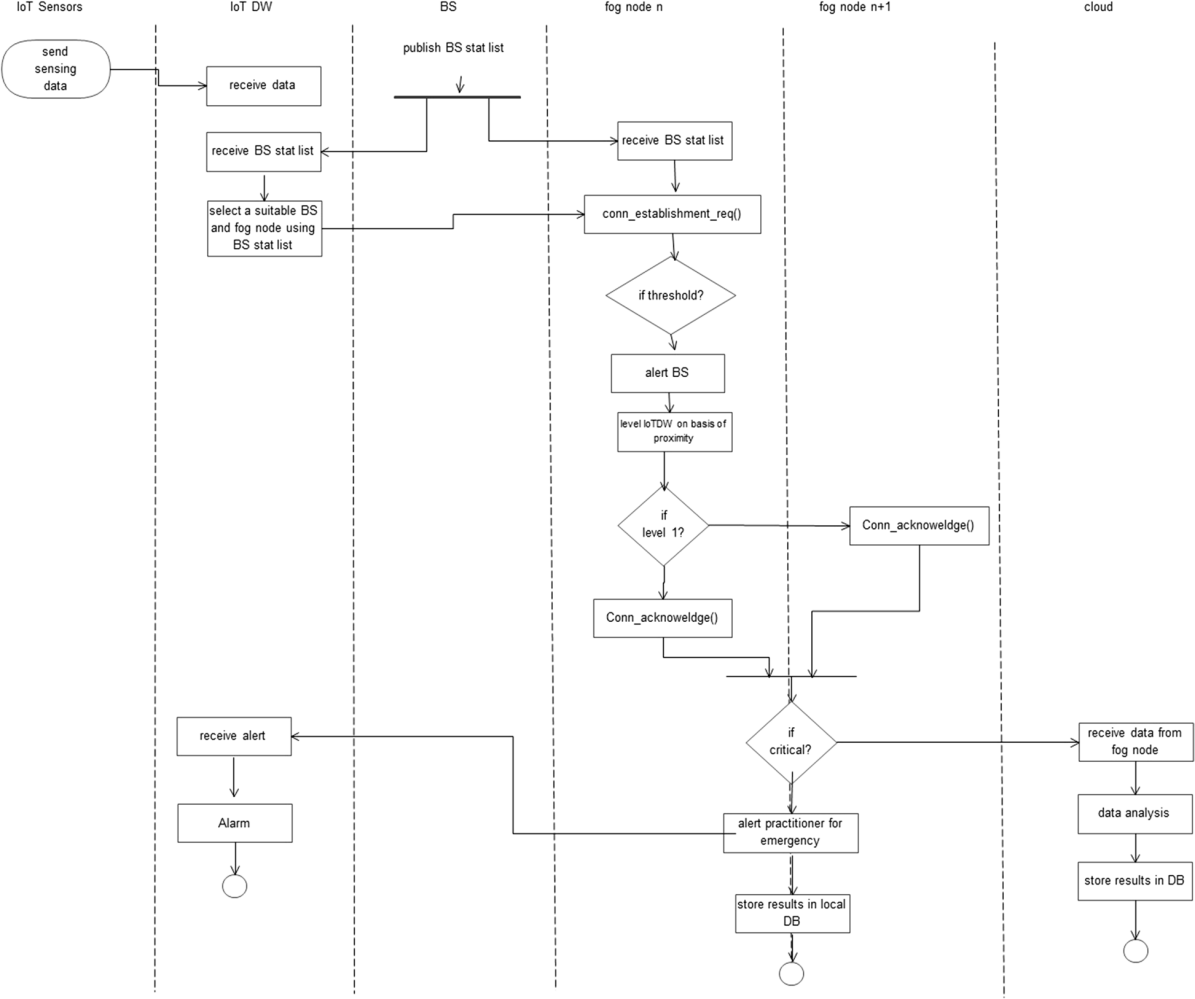
Activity diagram of the model

*Physical View:* The system is displayed in the physical view as seen by a systems engineer. This point of view focuses on the physical interactions and organizational structure of the software elements in the physical layer.

*Use Cases:* In this viewpoint, the architecture is described using a variety of examples or situations. The links between the items and processes are outlined in these situations. Developers are in charge of identifying certain requirements and designing a system to satisfy them because various systems may have distinct needs [35]. The two types of requirements are often distinguished as functional requirements and non-functional criteria that the system must satisfy.

## Simulation of the proposed architecture for verification

In this section, we simulate the proposed architecture to validate its working. Based on the activity flow and component diagram, the suggested architecture was modeled in Eclipse IDE using iFogSim toolkit [36]. The tool supports and simulates fog-based system architecture for analysis.

### Performance

This attribute refers to the fact of how long it takes to respond to an event. To this end, when using the system at peak time, nodes in the Fog and Cloud layer should handle high-quality services and, in the event of an overload of a node, requests are sent to lower-loaded nodes and thus processed in real-time.

### Reconfiguration

Fog-based IoT systems usually consist of dynamic configuration. In our framework, we describe systems that do not only include static structures but can react to events and specific requirements by the reconfiguration of the runtime of components and connectors, especially while managing the task assignment of incoming tasks at fog nodes.

**Fig. 7:**
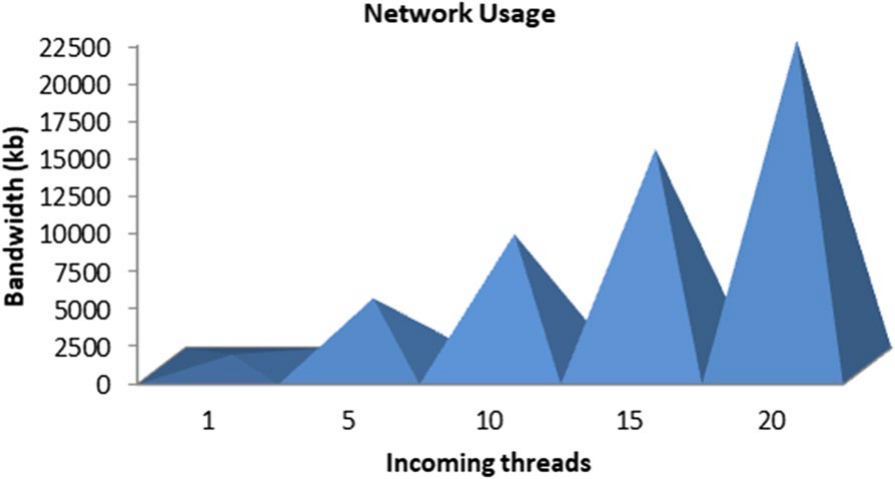
Network usage for various configurations

**Fig. 8:**
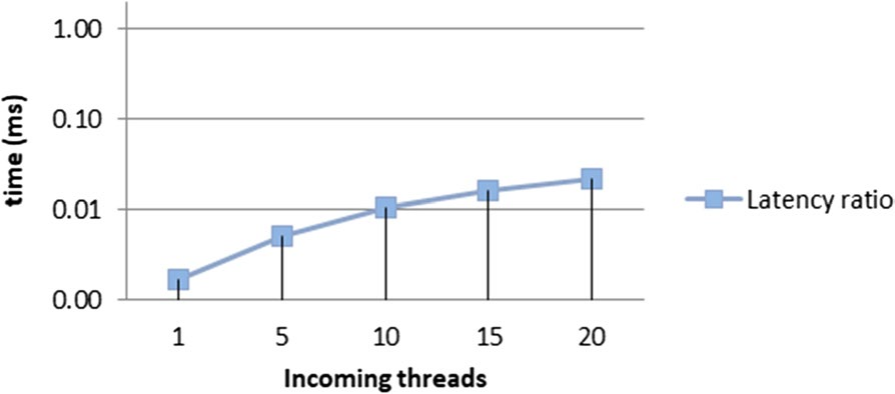
Latency ratio of the proposed architecture for various configurations

We formulated two algorithms (i) efficient scanning mechanism (ESM) and (ii) load balancing for real-time (LBRT) to observe the performance through latency changes in the environment. Network usage was also analyzed for the suggested architecture and techniques. The results depicted a 20%–25% improvement using the proposed architecture and algorithms. The outcomes for network usage and latency ratio are displayed in Figs. 7 and 8.

**Fig. 9:**
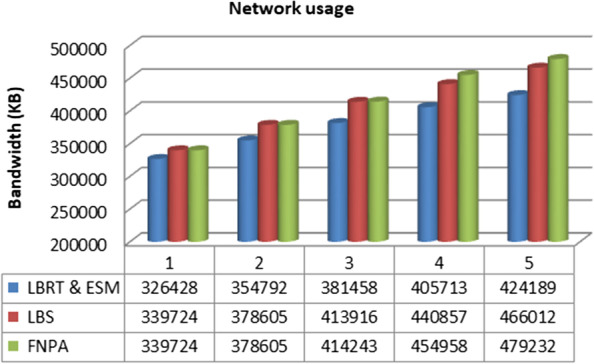
Comparison of network usage

**Fig. 10:**
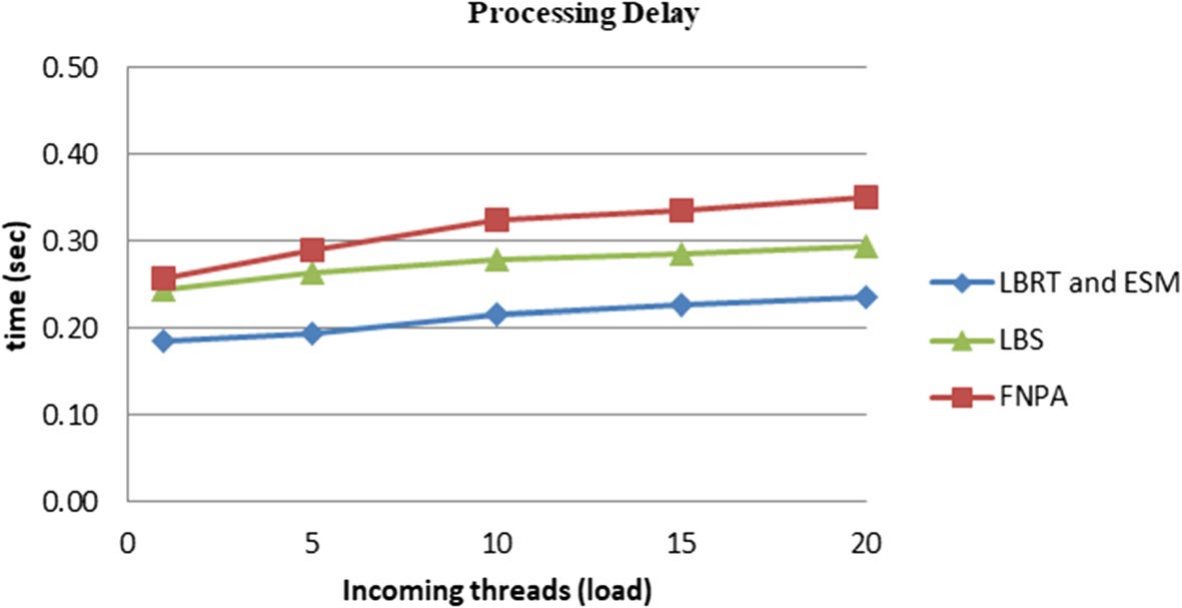
Comparison of processing delay

In Figs. 8 and 9, it can be seen that network usage, which is an important parameter for health monitoring systems, remains more or less measured and increases with the number of processing loads on the fog node. Similarly in the case of latency ratio, with the increasing number of threads, the latency increases in a controlled manner. While comparing the results with the schemes FNPA [8] and LBS [9] and other recent research, the suggested architecture and algorithms perform better as shown in Figs. 9 and 10 in terms of communication and transmission delay, load balancing, and task scheduling.

As seen in Figs. 9 and 10. The suggested software architecture along with devised algorithms ESM and LBRT, the results show notable improvement. The parameters used for the topology of fog-based environment are shown in Table 2.

**Table 2:**
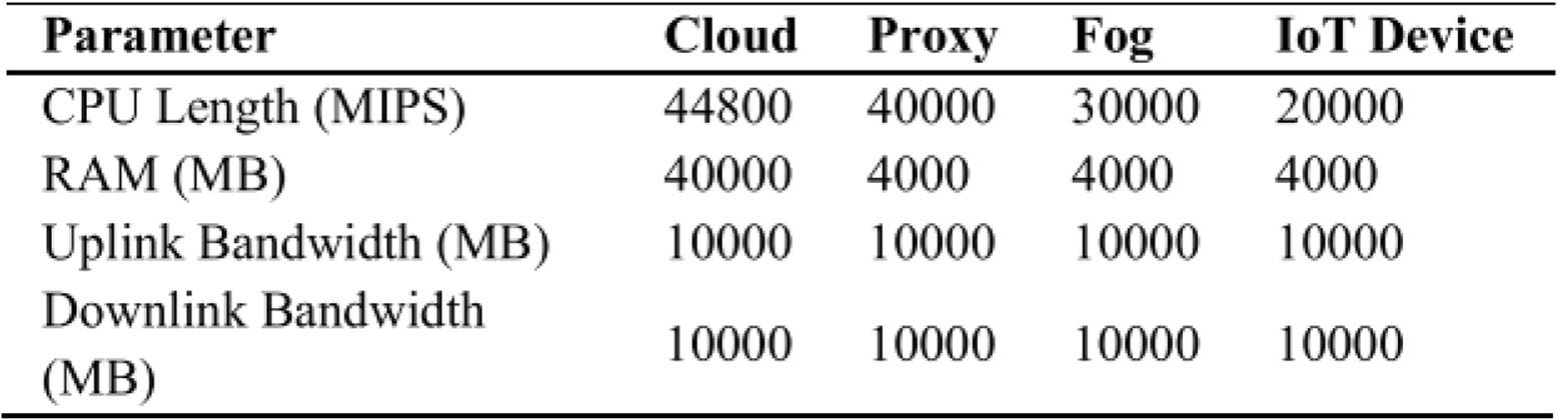
Topology parameters

## Conclusion

This article introduced hybrid architecture with built-in support for cloud and fog for IoT-based healthcare. The three layers of the proposed architecture are as follows: (1) the device layer, which includes wearable IoT sensors, and other end-user devices which connect to the IoTDW for sending data further; (2) the fog layer, which has a smart component for the dynamic decision to for improving fog efficiency; and (3) the cloud layer, which has resources for processing, storing, and retrieving data to offer services to end users. In addition, a novel task management technique is presented in this study that takes advantage of the fog layer to ensure continuous service delivery, data consistency, and load balancing. Model 4 + 1 demonstrates the proposed architecture’s layout and goes into great depth on its essential components. The suggested architecture was simulated using devised algorithms ESM and LBRT, which shows a notable improvement in comparison to the existing schemes in terms of network usage and latency. Future work in a similar domain may include novel security and privacy approaches such as blockchain and machine learning for handling health monitoring data. Moreover, patient and medical practitioner mobility aspects are also intended to be investigated in detail for future study.

## Data Availability

The supporting data can be provided on request.

## References

[1] Butpheng C, Yeh KH, Xiong H (2020). Security and privacy in IoT-cloud-based e-health systems—a comprehensive review. Symmetry.

[2] Fersi G (2021). Fog computing and internet of things in one building block: a survey and an overview of interacting technologies. Cluster Comput.

[3] Santos GL, Gomes D, Kelner J, Sadok D, Silva FA, Endo PT, Lynn T (2020). The internet of things for healthcare: optimising e-health system availability in the fog and cloud. Int J Comput Sci Eng.

[4] Safdar Z, Farid S, Qadir M, Asghar K, Iqbal J, Hamdani FK (2020). A novel architecture for the internet of things based E-health systems. J Med Imaging Health Inform.

[5] RahimiMoosavi S, NguyenGia T, Nigussie E, Rahmani AM, Virtanen S, Tenhunen H, Isoaho J (2016). End-to-end security scheme for mobility enabled healthcare internet. Future Gener. Comput. Syst..

[6] Alli AA, Alam MM (2020). The fog cloud of things: a survey on concepts, architecture, standards, tools, and applications. Internet of Things.

[7] Fan Q, Ansari N (2018). Towards workload balancing in fog computing empowered IoT. IEEE Transactions on Network Science and Engineering.

[8] Tun KN, Paing AMM (2020). Resource aware placement of IoT devices in fog computing. In 2020 international conference on advanced information technologies (ICAIT).

[9] Asghar A, Abbas A, Khattak HA, Khan SU (2021). Fog based architecture and load balancing methodology for health monitoring systems. IEEE Access.

[10] Farahani B, Firouzi F, Chang V, Badaroglu M, Constant N, Mankodiya K (2018). Towards fog-driven IoT eHealth: promises and challenges of IoT in medicine and healthcare. Future Gener. Comput. Syst..

[11] Woo MW, Lee J-W, Park K-H (2018). A reliable IoT system for personal healthcare devices. Future Gener. Comput. Syst..

[12] Verma P, Sood S-K (2018). Cloud-centric IoT based disease diagnosis healthcare framework. J. Parallel Distrib. Comput..

[13] Din S, Paul A (2019). Smart health monitoring and management system: toward autonomous wearable sensing for internet of things using big data analytics. Future Gener Comput Syst.

[14] Ullah S, Kim K, Kim HK, Imran M, Khan P, Tovar E, Ali F (2019). UAV-enabled healthcare architecture: issues and challenges. Future Gener. Comput. Syst..

[15] Kumari A, Tanwar S, Tyagi S, Kumar N (2018). Fog computing for healthcare 4.0 environment: opportunities and challenges. Comput Electr Eng.

[16] Dhanvijay MM, Patil CS (2019). Internet of things: a survey of enabling technologies in healthcare and its applications. Comput Netw.

[17] Abbasi M, Mohammadi-Pasand E, Khosravi MR (2021). Intelligent workload allocation in IoT–fog–cloud architecture towards mobile edge computing. Comput Commun.

[18] Mshali H, Lemlouma T, Moloney M, Magoni D (2018). A survey on health monitoring systems for health smart homes. Int J Ind Ergon.

[19] Hazra A, Adhikari M, Amgoth T, Srirama SN (2021). Stackelberg game for service deployment of IoT-enabled applications in 6Gaware fog networks. IEEE Internet Things J.

[20] Hazra A, Adhikari M, Amgoth T, Srirama SN (2020). Joint computation offloading and scheduling optimization of IoT applications in fog networks. IEEE Trans Netw Sci Eng.

[21] Rahmani A-M, Gia T-N, Negash B, Anzanpour A, Azimi I, Jiang M, Liljeberg P (2018). Exploiting smart E-health gateways at the edge of healthcare internet-of-things: a fog computing approach. Future Gener. Comput. Syst..

[22] Tuli S, Basumatary N, Gill SS, Kahani M, Arya RC, Wander GS, Buyya R (2020). HealthFog: “an ensemble deep learning based smart healthcare system for automatic diagnosis of heart diseases in integrated IoT and fog computing environments.”. Future Gener. Comput Syst.

[23] Constant N, Borthakur D, Abtahi M, Dubey H, Mankodiya K (2017) Fog-assisted wiot: a smart fog gateway for end-to-end analytics in wearable internet of things. arXiv preprint arXiv:1701.08680. https://arxiv.org/abs/1701.08680

[24] Ali F, El-Sappagh SH, Riazul Islam SM, Ali A, Attique M, Imran M, Kwak K-S (2021). An intelligent healthcare monitoring framework using wearable sensors and social networking data. Future Gener. Comput. Syst..

[25] Kaur A, Sood S-K (2021) Cloud-fog assisted energy efficient architectural paradigm for disaster evacuation. Inf Syst. 10.1016/j.is.2021.101732

[26] Alam MD, ShirajumMunir MD, Zia Uddin MD, ShamsulAlam MD, Nguyen Dang T, Hong CH (2019). Edge-of-things computing framework for cost-effective provisioning of healthcare data. J. Parallel Distrib. Comput..

[27] Rahmani AM, Babaei Z, Souri A (2020). Event-driven IoT architecture for data analysis of reliable healthcare application using complex event processing. Clust Comput.

[28] Sahoo PK, Mohapatra SK, Wu SL (2018). SLA based healthcare big data analysis and computing in cloud network. J Parallel Distrib Comput.

[29] Sarrab M (2021). Assisted-fog-based framework for IoT-based healthcare data preservation. Int J Cloud Appl Comput (IJCAC).

[30] Chudhary R, Sharma S (2021). Fog-cloud assisted framework for heterogeneous internet of healthcare things. Procedia Comput Sci.

[31] Mahini H, Rahmani AM, Mousavirad SM (2021). An evolutionary game approach to IoT task offloading in fog-cloud computing. J Supercomput.

[32] Alazab M, Khan LU, Koppu S, Ramu SP, Iyapparaja M, Boobalan P et al (2022) Digital twins for healthcare 4.0-recent advances, architecture, and open challenges. IEEE Consumer Electronics Magazine

[33] Chengoden, R., Victor, N., Huynh-The, T., Yenduri, G., Jhaveri, R. H., Alazab, M., ... & Gadekallu, T. R. (2022). Metaverse for Healthcare: A Survey on Potential Applications, Challenges and Future Directions. arXiv preprint arXiv:2209.04160

[34] Chihoub EH, Ibrahim S, Antoniu G, Perez SM, Sakr S, Gaber M (2014). Consistency management in cloud storage systems. Advances in data processing techniques in the era of big data.

[35] Andrade E, Nogueira B, Farias Júnior ID, Araújo D (2021). Performance, and Availability Trade-Offs in Fog–Cloud IoT Environments. J Netw Syst Manage.

[36] Gupta H, Vahid Dastjerdi A, Ghosh SK, Buyya R (2017). iFogSim: a toolkit for modeling and simulation of resource management techniques in the internet of things, edge and fog computing environments. Software: Practice and Experience.

